# Exploitation of wasted bread as substrate for polyhydroxyalkanoates production through the use of *Haloferax mediterranei* and seawater

**DOI:** 10.3389/fmicb.2022.1000962

**Published:** 2022-09-21

**Authors:** Marco Montemurro, Gaia Salvatori, Sara Alfano, Andrea Martinelli, Michela Verni, Erica Pontonio, Marianna Villano, Carlo Giuseppe Rizzello

**Affiliations:** ^1^Department of Soil, Plant and Food Science, University of Bari Aldo Moro, Bari, Italy; ^2^Department of Chemistry, Sapienza University of Rome, Rome, Italy; ^3^Research Center for Applied Sciences to the Safeguard of Environment and Cultural Heritage (CIABC), Sapienza University of Rome, Rome, Italy; ^4^Department of Environmental Biology, Sapienza University of Rome, Rome, Italy

**Keywords:** bioplastic, *Haloferax mediterranei*, wasted bread, bioprocessing, seawater, polyhydroxyalkanoates (PHA)

## Abstract

The use of the halophile microorganism *Haloferax mediterranei*, able to synthesize poly(hydroxybutyrate-hydroxyvalerate) (PHBV), is considered as a promising tool for the industrial production of bioplastic through bioprocessing. A consistent supplementation of the growth substrate in carbohydrates and minerals is overall necessary to allow its PHBV production. In this work, wasted bread was used as substrate for bioplastic production by microbial fermentation. Instead of the consistent and expensive minerals supplement required for *Hfx. mediterranei* DSM1411 growth, microfiltered seawater was added to the wasted bread-derived substrate. The suitable ratio of wasted bread homogenate and seawater, corresponding to 40:60, was selected. The addition of proteases and amylase to the bread homogenate promoted the microbial growth but it did not correspond to the increase of bioplastic production by the microorganism, that reach, under the experimental conditions, 1.53 g/L. An extraction procedure of the PHBV from cells, based on repeated washing with water, followed or not by a purification through ethanol precipitation, was applied instead of the conventional extraction with chloroform. Yield of PHBV obtained using the different extraction methods were 21.6 ± 3.6 (standard extraction/purification procedure with CHCl_3_:H_2_O mixture), 24.8 ± 3.0 (water-based extraction), and 19.8 ± 3.3 mg PHAs/g of wasted bread (water-based extraction followed by ethanol purification). Slightly higher hydroxyvalerate content (12.95 vs 10.78%, w/w) was found in PHBV obtained through the water-based extraction compared to the conventional one, moreover, the former was characterized by purity of 100% (w/w). Results demonstrated the suitability of wasted bread, supplemented with seawater, to be used as substrate for bioplastic production through fermentation. Results moreover demonstrated that a solvent-free extraction, exclusively based on osmotic shock, could be used to recover the bioplastic from cells.

## Introduction

Eighty percent of the plastic manufactured worldwide, equivalent to 3.5 billion tons of carbon (based on approximately 60% carbon content of plastics), is not recycled or re-used in other ways (Blank et al., 2020), thus drastically increasing worries over plastic pollution. Petroleum-derived plastics dispersed in the environment, oceans included, might take over 100 years to be mineralized (Kumar et al., 2020). Plastic recycling is the most appropriate way of waste management but the process is tremendously slow, hindered by the properties of different plastics and limited due to the presence of heterogeneous materials and additive substances such as coverings, fillers, and coloring materials (Kumar et al., 2020). With the European Union Directive EU 2019/904 of May 21, 2019, the European Parliament finally adopted the restriction on the use of a variety of disposable plastic objects, with the exception of those made with natural polymers that have not been chemically changed.

Bioplastics are a sustainable alternative to conventional plastics since they are biodegradable and/or derived from renewable sources (Imre et al., 2019; Sidek et al., 2019). In recent years, the search for eco-friendly plastic materials has been intense, resulting in a growing production of disposable objects made from bioplastics. Among the different macromolecules showing suitable technological properties to be potentially considered as candidates to replace petroleum-derived plastics, polyhydroxyalkanoates (PHAs) are under intense investigation. PHAs are linear polyesters that could be synthesized and stored as an intracellular reserve of energy and carbon source by a variety of bacteria, which help the microorganisms survive under environmental stress conditions (Jendrossek, 2009; Han et al., 2015). It was reported that, under specific conditions, PHAs can be accumulated up to the 90% of the total cell dry weight (Patel et al., 2021). PHAs have been discovered as attractive candidates for the manufacturing of bio-based plastics due to their combined biodegradability, biocompatibility, and thermoplasticity features (Han et al., 2017). The broad biotechnological applications of PHAs affect several sectors, and include aquaculture, agricultural and biomedical uses (Kalia et al., 2021). Although PHAs offer enormous market potential, exorbitant production costs currently prevent their widespread use (Albuquerque and Malafaia, 2018), nevertheless the use of biowaste as low-cost feed and efficient PHAs-producers has been already reported to be helpful in the economic PHAs production (Patel et al., 2012). Primarily PHA homo-polymers production such polyhydroxybutyrate (PHB) is well established by PHAs-producers microrganisms. However, the PHA co-polymers production, such as that of poly(hydroxybutyrate-hydroxyvalerate (P(3HB-co-3HV), PHBV) is desirable due to the optimal physical properties compared to PHB (Kumar et al., 2014).

The utilization of halophilic bacteria, a wide microbial group that can thrive in hypersaline conditions, is garnering the attention of scientific and industrial researchers as a sustainable tool for manufacturing PHAs (Yin et al., 2015). The ability of halophiles to adapt to harsh circumstances increases their interest for PHAs production. High salinity requirements drastically minimize the likelihood of microbiological contamination (Kourmentza et al., 2017), allowing bioprocessing to be conducted also without expensive substrate sterilization. Due to the high intracellular osmotic pressure, microbial cells can be rapidly lysed in demineralized water, substantially decreasing the cost of PHAs recovery (Quillaguamán et al., 2010), and the environmental impact related to the disposal of the organic solvents required for conventional extraction.

When dealing with pure microbial cultures, PHAs production typically requires substrates with a high carbohydrate content. Therefore, waste biomass from the food sector, including bread and other bakery waste, might serve as a source of nutrients for the manufacturing of bioplastics. Melikoglu and Webb (2013) estimated that the daily global bread waste corresponds to hundreds of tons, nevertheless the quantification results complicated since largely variable among the different countries. Overall, bread waste accounts for up to 6–7% of the quantity delivered by suppliers (Cicatiello et al., 2017) and represents 27–30.6% of the total food waste mass in many European countries (Soni et al., 2022). Losses varied from 4 kg/person/year in Finland and Italy to double values in other countries, such as in South Africa (6–8 kg/person/year) (Goryńska-Goldmann et al., 2020). Sweden’s annual bread waste was estimated to be 80,410 tons, corresponding to 8.1 kg per person (Goryńska-Goldmann et al., 2020). Current data tend to be underestimated since the amount of bread returned to the manufacturers before the sell-by date (e.g., 8–10% of bread production in Germany), is often not considered (Melikoglu and Webb, 2013; Cicatiello et al., 2017).

Recently, different studies investigated the use of *Haloferax mediterranei* for PHAs production by using food waste as substrate (Raho et al., 2020; Wang and Zhang, 2021). *Hfx. mediterranei*, in particular, is able to synthesize the PHBV copolymer (Han et al., 2015) which, compared to the PHB homopolymer, exhibits improved physical properties, which are function of the HV content (Dalton et al., 2022).

The potential of wasted bread to be used as substrate for *Hfx. mediterranei* growth and bioplastic synthesis was investigated in this work. Moreover, considering that the supplementation of the substrate with several minerals and trace elements is needed to guarantee *Hfx. mediterranei* growth (Torreblanca et al., 1986; Koller et al., 2007), thus increasing bioplastic production costs, the use of microfiltered seawater as cheap source of micronutrients in bread-derived substrate was proposed. Results demonstrated the suitability of wasted bread/seawater substrate to be used for bioplastic production through fermentation. A solvent-free extraction to recover the bioplastic from cells was also set-up.

## Materials and methods

### Raw materials and enzymes

Wasted bread was collected in local supermarket (Bari, Italy) and ground into small crumbs (<1 mm) with an Ika-Werke M20 laboratory grinder (GMBH, Staufen, Germany). The proximal composition of bread, made with wheat flour, was: moisture, 11.84%; proteins, 12.12%; fat, 1.38%; carbohydrates, 70.12%; and dietary fibers, 4.54%.

Pepsin (from porcine gastric mucosa, 400 U/mg of protein) (Sigma-Aldrich, United States), α-amylase (from porcine pancreas, 5 U/mg) (Sigma-Aldrich, United States), and the protease Veron PS (from *Aspergillus oryzae*, 227 U/g) (AB enzymes, Germany) were used for the treatment of the wasted bread; α-amylase and α-amyloglucosidase (from *Aspergillus niger*, 120 U/mg) (Sigma-Aldrich, USA) were used for the hydrolysis of the starch into glucose before quantification.

### Microorganism propagation

*Hfx. mediterranei* DSM1411 was purchased from the DSMZ collection (Braunschweig, Germany) and cultivated in the culture medium 372 prepared according to the technical data sheet reported on the DSMZ website. The cell culture was propagated daily using a 20% inoculum coming from the broth culture of the day before and incubated at 37°C for 24 h under stirring at 150 rpm. The growth of *Hfx. mediterranei* DSM1411 (initial cell density of 7 log10 cfu/mL) in the culture medium 372 (12 mL in Erlenmeyer flask of 50 mL volume) was evaluated by cell density at 600 nm (Fang et al., 2010) and by microscopic count obtained using the Thoma cell counting chamber. The growth of *Hfx. mediterranei* DSM1411 was then evaluated every 4 h during the fermentation at 37°C in stirring condition (150 rpm) for 24 h. The correlation between optical density and cell count was then calculated.

### Wasted bread substrate

#### Bread: Water ratio

Aiming at the preparation of a suitable substrate for *Hfx. mediterranei* growth, suspensions of ground bread at different percentages (10, 15, and 20% w/v) in demineralized water were prepared and tested as substrates for microbial cultivation. Mixtures were incubated for 60 min at 4°C under stirring condition (200 rpm) before enzymatic treatment, to obtain a complete hydration and a homogeneous distribution of the bread particles.

#### Enzymatic treatments

Pepsin was added to the substrate at the concentration of 3 mg/g of bread (after pH adjustment at 2.5 with 0.5 M HCl), in a treatment carried out at 37°C for 3 or 6 h, while α-amylase was added at 5 mg/g of bread (after pH adjustment at 6.9 with 0.2 M NaOH) in a treatment carried out at 37°C for 3 h. The enzymatic treatments were carried out with pepsin alone (3 and 6 h of incubation) and with pepsin and α-amylase sequentially (3 + 3 and 6 + 3 h of incubation).

At the end of enzymatic treatment, the insoluble material was removed by centrifugation at 12,800 × *g* for 10 min at 4°C, recovering the supernatant. Starch and free glucose concentration into the supernatant were quantified as described by De Angelis et al. (2007). In particular, starch was previously hydrolyzed adding 100 μl of α-amylase (200 U/mL) to 2 mL of supernatant and incubating for 1 h at 37°C. After, 3 mL of 0.4 M sodium acetate buffer (pH 4.75) and 80 μl of amyloglucosidase (140 U/mL) were added to totally hydrolyze the starch into glucose after 45 min at 60°C. The sum of the free glucose and the hydrolyzed starch was evaluated by using the D-Glucose Assay Kit (GOPOD Format, Megazyme, Ireland) according to manufacturer’s instruction. The ratio (% w/w) of glucose solubilized during substrate preparation on the total carbohydrates content was calculated and expressed as Glucose Yield (GY).

The protease Veron PS (AB enzymes, Germany) was also used, instead of pepsin, aiming at replacing a laboratory-grade enzyme with a commercial and cheaper formulation, intended for industrial uses. Veron PS was used at dosages recommended by the manufacturer and 2.5 and 5 folds higher (40, 100, and 200 g/100 kg of bread) without pH adjustment of the bread homogenate. A substrate not subjected to enzymatic treatment and pH adjustment incubated for 1 h at 4°C was also included in the experimental design.

#### Seawater supplementation

Microfiltered (MF-Millipore membrane filter, 0.45 μm) seawater collected from Bari (Italy) was mixed to the wasted bread homogenate at percentages of 45, 60, and 75% (v/v). Seawater was characterized for calcium content of 40.6 ± 1.16 mg/100 mL; magnesium, 124.9 ± 0.63 mg/100 mL; potassium, 41.9 ± 5.60 mg/100 mL; sodium 1,064.00 ± 4.60 mg/100 mL, and chloride, 2419.00 ± 25.70 mg/100 mL, as previously reported by De Bellis et al. (2020).

#### *Hfx. mediterranei* growth test

All the substrates were sterilized at 121°C for 15 min and separated from the insoluble residue by centrifugation at 12,800 × *g* for 10 min at 4°C, recovering the supernatant. Substrates were then filtered with MF-Millipore membrane filter (0.45 μm pore size, Merck KGaA, Darmstadt, Germany), added with 160 g/L of NaCl, and adjusted to pH 7.2 with 1M ammonia solution.

*Hfx. mediterranei* DSM1411 growth tests were carried out in 50 mL sterile Erlenmeyer flask containing 12 mL of wasted bread substrate and 2 mL of 24-h broth culture inoculum. The evaluation of microbial growth was performed spectrophotometrically at 600 nm after 24, 48, and 72 h of incubation at 37°C in stirring condition (200 rpm).

### Bioplastic production

Microbial biomass, raw and purified PHBV in wasted bread substrates fermented for 72 h at 37°C were determined as previously described by Raho et al. (2020) with some modification at 1L-Erlenmeyer flask level. In particular, bioplastic production was tested on the 15% wasted bread substrates obtained: (i) by the 6 h-pepsin treatment (P6); (ii) by the 200 g/100 kg Veron PS treatment (PS200); (iii) without enzymatic treatment (NE). In any case, wasted bread substrates were mixed with microfiltered seawater in the ratio 40:60, added with 160 g/L of NaCl, and adjusted to pH 7.2 with 1M ammonia solution.

The exhausted substrate was separated from the cells by centrifugation (12,800 × *g* for 10 min at 4°C). Then, to prevent cellular lysis, the cells were washed with a 160 g/L NaCl solution and centrifuged again. The cell pellet (cell dry matter, CDM) was dried at 60°C for at least 24 h and weighed. PHBV was then extracted from dry biomass through resuspension of CDM in 20 mL/g of deionized water until complete solubilization. The supernatant was discarded after centrifugation (12,800 × *g* for 20 min at 4°C) and the pellet was resuspended in 30 mL/g of CDM in CHCl_3_:H_2_O mixture (1:1 v/v). The PHBV-containing organic phase was separated and centrifuged again at 12,800 × *g* for 10 min at 4°C to remove impurities recovering the raw bioplastic after evaporation of CHCl_3_. The raw bioplastic was weighed and the polymer content (PC) (i.e., ratio of PHAs contained in the CDM) was calculated according to the following formula:


$$PC=\left[\text{PHBV}\;\left(\frac{\text{mg}}{\text{L}}\right)÷\text{CDM}\;\left(\frac{\text{mg}}{\text{L}}\right)\right]\times 100$$

PHBV was dissolved in CHCl_3_ and cold ethanol was added in a ratio 1:10 v/v aiming at the purification of the bioplastic. Purified PHBV (corresponding to the precipitate) was recovered with a centrifugation (12,800 × *g* for 10 min at 4°C) and characterized (pPBHV).

The polymer yield (PY) was expressed as PHBV (mg/L) produced from the substrate (g/L of wasted bread).


$$PY=\left[\text{PHBV}\;\left(\frac{\text{mg}}{\text{L}}\right)÷\mathrm{wasted}\mathrm{bread}\;\left(\frac{\text{g}}{\text{L}}\right)\right]$$

The overall amount of polymer recovered was expressed as g/L of the liquid substrate employed for the fermentation process.

### Scale-up of bioplastic production in bioreactor

The wasted bread substrate considered suitable for the PHBV sustainable production was chosen according to the bioplastic yield and the lowest use of additional input. It corresponded to the 15% wasted bread homogenate obtained without enzymatic treatment and incubated for 1 h at 4°C, mixed with microfiltered seawater in the ratio 40:60, sterilized at 121°C for 15 min, filtered with MF-Millipore membrane filter, added with 160 g/L of NaCl, and adjusted to pH 7.2 with 1M ammonia solution.

Such substrate was prepared and fermented with *Hfx. mediterranei* DSM1411 in a 3 L-bioreactor (Diachrom Biotechnology, Switzerland) with automatic control of pH and temperature, which were set at values of 7.2 ± 0.01 and 37 ± 0.1°C, respectively. Fermentation lasted 72 h and it was carried out under controlled stirring at 400 rpm, using half of the available volume of the bioreactor, to promote aerobic conditions. Foaming was inhibited by the automatic addition of Antifoam A (Sigma, Milan, Italy). At the end of the incubation, the cells were recovered as described above.

### Alternative poly(hydroxybutyrate-hydroxyvalerate) extraction

**FIGURE 1:**
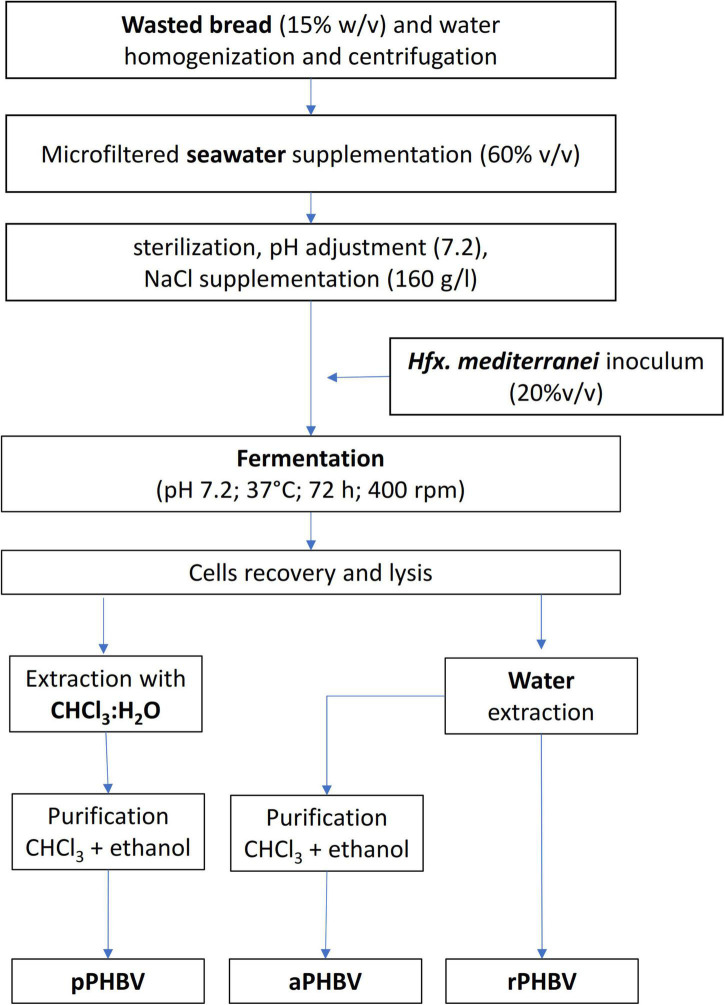
Flow chart of the PHBV production and recovery from the wasted bread/seawater substrate: pPHBV, obtained from the CHCl_3_:H_2_O separation and the precipitation with cold ethanol; rPHBV, obtained by the replacement of the CHCl_3_:H_2_O separation with repeated washing with water; aPHBV, obtained by the replacement of the CHCl_3_:H_2_O separation with repeated washing with water but followed by solubilization in CHCl_3_ and purification with cold ethanol.

In order to limit the use of chloroform, the CDM resuspended in deionized water for lysis was washed with deionized water (20 mL/g) and centrifuged (with supernatant removal) for 8 times, instead of the separation in CHCl_3_:H_2_O, aiming at promoting the complete release of PHBV by osmotic shock (Figure 1). The sample (coded as raw PHBV, rPHBV) was then characterized. After the repeated washing, an aliquot of the rPHBV pellet was further purified (by resuspension in 10 mL of chloroform per g of starting CDM), precipitated by adding 10 mL of ethanol per mL of chloroform, and centrifuged as described before (Figure 1). Such sample was coded as aPHBV (PHBV from alternative extractions).

### Characterization of poly(hydroxybutyrate-hydroxyvalerate)

Three different samples were characterized: (i) pPHBV obtained from the CHCl_3_:H_2_O separation and the precipitation with cold ethanol; (ii) rPHBV obtained by the replacement of the CHCl_3_:H_2_O separation with repeated washing with water; (iii) aPHBV obtained by the replacement of the CHCl_3_:H_2_O separation with repeated washing with water followed by solubilization in CHCl_3_ and precipitation with cold ethanol. The processes used to recover the samples is reported in Figure 1.

The determination of the PHBV purity and composition in all extracted samples has been performed by means of gas chromatography (GC) analysis. For this purpose, approximately 4 mg of dried sample were suspended in 2 mL of an acidified (3% v/v H_2_SO_4_) methanol solution in a screw-capped test tube. Subsequently, 1 mL of chloroform was added, and samples were kept at 100°C for 4 h to allow for PHAs methanolysis, according to the method described elsewhere (Braunegg et al., 1978). After cooling at room temperature, 1 mL of distilled water was added in order to obtain an aqueous and an organic phase, with the latter containing the 3-hydroxyacyl-methyl esters that were quantified by GC-FID Perkin Elmer 8410 (Perkin Elmer, MA, USA).

The relative abundance of 3HB and 3HV monomers was determined using a commercial P(3HB-co-3HV) copolymer with a 3HV content of 5 (% w/w) (Sigma-Aldrich, Milan, Italy) as reference standard. The PHBV content in each sample, referred to PHBV purity (as %, w/w), was calculated as the ratio between the measured PHBV (as sum of the 3HB and 3HV monomers) and the amount of extracted dried biomass used for the analysis. The 3HV content in the stored PHBV copolymers was calculated as the ratio between 3HV and (3HB + 3HV) monomers (expressed as %, w/w). For each sample analysis were performed at least in duplicate.

FT-IR spectra in attenuated total reflection mode (ATR) were acquired by using a Thermo Nicolet 6700 instrument (Thermo Scientific, MA, USA), equipped with a Golden Gate diamond single reflection device (Specac LTD, England). The spectra were collected co-adding 200 scans at a resolution of 4 cm^–1^ in the range 4,000–600 cm^–1^.

The thermal stability of the obtained PHBV copolymers was investigated by thermogravimetric analysis (TGA), using a Mettler TG 50 thermobalance equipped with a Mettler TC 10 A processor. Approximately 4 mg of sample were used for the analysis. All measurements were carried out under nitrogen flow from 25°C to 500°C, at a heating rate of 10°C min^–1^. The thermal behavior of samples was characterized by differential scanning calorimetry (DSC) by using a Mettler Toledo DSC 822e (Mettler Toledo, Ohio, USA). About 4–6 mg of sample were put in aluminum pans and subjected to a temperature program consisting of three different scans under nitrogen flow (30 mL min^–1^). A first heating scan was conducted from 25°C to 190°C with a linear increase of 10°C min^–1^ to melt the polymer and erase its previous thermal history. Then, the sample was cooled from 190°C to −70°C at 30°C min^–1^ and heated again from −70°C to 190°C at 10°C min^–1^, to evaluate the intrinsic thermal properties of samples.

### Statistical analysis

The data were analyzed using one-way ANOVA followed by Tukey’s test to detect significant different values (*P* < 0.05) by Statistica 12.0 software. The Pearson correlation coefficient was used to evaluate the correlation between spectrophotometric and microscopical cell counting.

## Results

### Preliminary wasted bread substrate preparation

The simple resuspension of the wasted bread in water (up to 200 g/L) led to the release of amount of carbohydrates (evaluated as glucose equivalent) lower than 32 g/L, with a GY decreasing at concentration of bread higher than 150 g/L (Table 1). Therefore, 150 g/L of bread was chosen as starting condition to investigate the effect of the enzymatic treatments. As expected, the use of enzymes led to a significant increase in glucose extraction yield. In particular, pepsin increased the GY values, especially when it was used as a pre-treatment before α-amylase (Table 1). In these conditions, the quantified glucose in solution increased of the 47 (pepsin for 3 h and α-amylase treatment) and 66 % (pepsin for 6 h and α-amylase treatment) compared to the sample obtained without enzymes addition (Table 1).

**TABLE 1:** Carbohydrates release expressed as glucose (g/L) and Glucose Yield (GY, %) in wasted bread homogenates obtained without enzyme treatment (incubation 1 h at 4°C), after pepsin treatment at 37°C for 3 or 6 h (P3 or P6, respectively) (3 mg/g of bread, pH solution of 2.5), α-amylase treatment at 37°C for 3 h (A) (5 mg/g of bread, solution pH of 6.9), or after sequential treatment with both the enzymes (P + A).

Bread (g/L)	Enzymes	Glucose equivalent (g/L)	GY (%)
100	–	21.6 ± 1.0^a^	30.8^b^
150	–	32.1 ± 1.2^b^	30.5^b^
200	–	37.2 ± 1.1^c^	26.5^a^
150	A	35.0 ± 1.1^d^	33.3^d^
150	P3	39.3 ± 1.8^c^	37.4^c^
150	P3+A	47.3 ± 1.2^e^	45.0^e^
150	P6	45.7 ± 1.7^e^	43.4^e^
150	P6+A	53.4 ± 2.1^f^	50.8^f^

The data are the means of three independent experiments ± standard deviations (*n = 3*).

*^a–f^*Values in the same column with different superscript letters differ significantly (*p* < 0.05).

### Estimation of the *Hfx. mediterranei* growth

The growth of *Hfx. mediterranei* DSM1411 in 372 media was both monitored by the Thoma cell counting chamber and spectrophotometrically. High positive correlation was found between the data obtained with the two methods (*R* = 0.9946, *p* < 0.001). On the basis of this result, the following linear equation was elaborated and applied for the determination of the cell density in all the further experiments on wasted bread derived substrates:


y=27.474×A600

where y represents the cell density to be multiplied by 10^7^ cfu/mL, while A600 represents the value of absorbance at 600 nm with A600 > 0.020.

### Growth in wasted bread substrate and seawater supplementation

**FIGURE 2:**
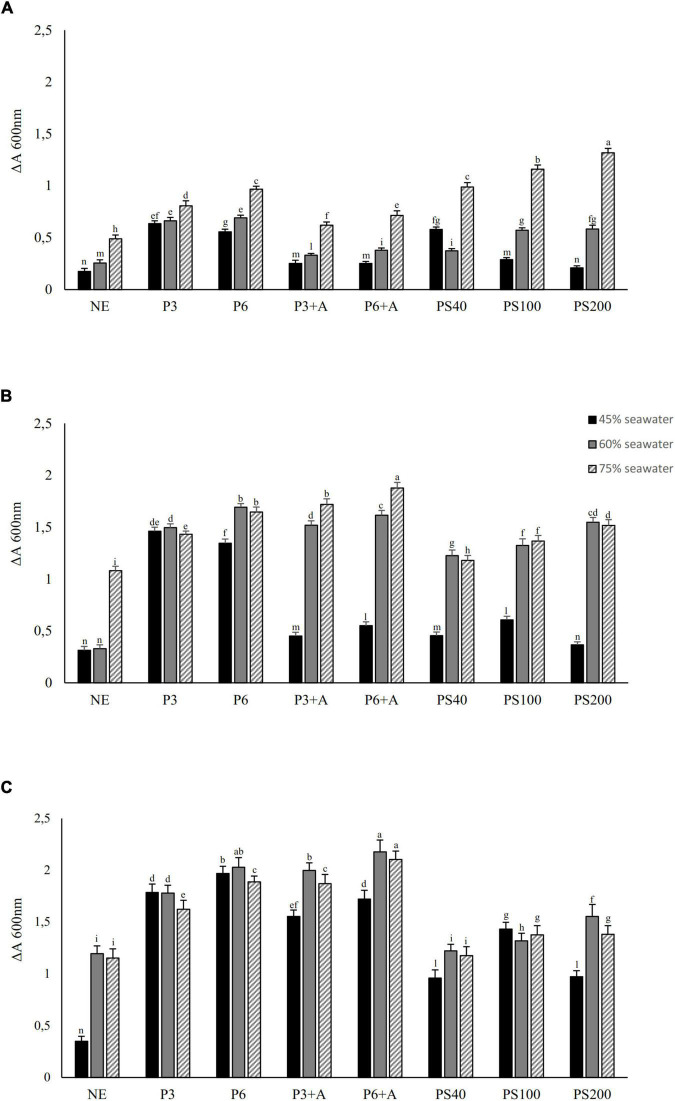
Growth tests of *Hfx. mediterranei* DSM1411 (values expressed as absorbance, ΔA at 600 nm) on different wasted bread substrates obtained without enzyme treatment (incubation 1 h at 4°C), after pepsin treatment at 37°C for 3 or 6 h (P3 or P6, respectively) (3 mg/g of bread, pH solution of 2.5), α-amylase treatment at 37°C for 3 h **(A)** (5 mg/g of bread, solution pH of 6.9), or after sequential treatment with both the enzymes (P + A). Bread homogenates subjected to a treatment (37°C for 6 h) with commercial protease formulation Veron PS was also included (40, 100, and 200 g/100 kg bread; respectively PS40, PS100, and PS200). A wasted bread homogenate was prepared without enzymes (NE). Seawater was added at 45, 60, and 75%vol/vol. All substrates were added with 160 g/L of NaCl and brought to pH 7.2 with 1M ammonia solution before inoculum of 7 log10 cfu/mL. Cell density was detected as a difference between the start of incubation (37°C in stirring condition) and 24 **(A)**, 48 **(B)**, or 72 h **(C)**. The data are the means of three independent experiments ± standard deviations (*n* = 3). *^a–n^*Values with different superscript letters differ significantly (*p* < 0.05).

Considering the *Hfx. mediterranei* requirements in minerals (Torreblanca et al., 1986; Koller et al., 2007), microfiltered seawater was mixed at different percentages with the wasted bread substrate. A stunted growth of the microorganism was observed in wasted bread substrate supplemented with lower than 40% seawater (data not shown), while mixtures containing 45, 60 and 75% (v/v) seawater corresponded to relevant increases in *Hfx. mediterranei* cell density (Figure 2).

The growth of *Hfx. mediterranei* at 37°C was monitored (until 72 h) in substrates obtained by different combination of enzymatic treatments and seawater supplementation (Figure 2). Longer incubation time did not lead to significant increase of the microbial cell density.

Compared to the substrate obtained without the enzymatic treatment, pepsin supplementation led to a significant higher microbial cell density (Figure 2). In particular, the pepsin treatment lasting 6 h (P6) significantly improved the absorbance compared to the 3 h-treatment. Slightly, but not significant, differences of the absorbance were found between treatments with pepsin for 6 h and pepsin for 6 h followed by amylase when 60% of seawater was used. These conditions corresponded to the highest absorbance at 72 h of incubation (2.028 ± 0.092 and 2.174 ± 0.115, respectively) (Figure 2). When Veron PS was used instead of pepsin, cell densities were significantly lower. Among the three concentrations of Veron PS tested, treatment with 200 g/100 kg led to the higher microbial growth.

Overall, incubation prolonged for 72 h and the use of seawater at 60% gave the best results of all the conditions tested.

### Scale-up of bioplastic production

PHBV synthesis was investigated in three different process conditions at 1 L-Erlenmeyer flask level:

i) fermentation of the substrate obtained with 6 h-pepsin treatment (P6), which corresponded to the highest growth of the microorganism among all the conditions considered;
ii) fermentation of the substrate treated with 200 g/100 kg of Veron PS (PS200), which corresponded to the highest growth when the enzyme intended for industrial application was used;
iii) fermentation of the substrate obtained without enzymatic treatment (NE), as control.

In each case, fermentation lasted 72 h at 37°C and seawater was mixed at 60% v/v.

**FIGURE 3:**
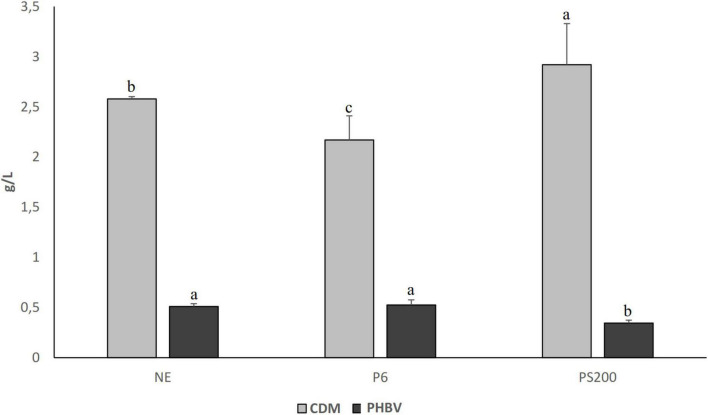
Cell dry matter (CDM) and PHBV production by *Hfx. mediterranei* DSM1411 in different wasted bread derived substrates produced using pepsin for 6 h (P6), Veron PS at the concentration of 200 g/100 kg (PS200) and without enzymes (NE) and diluted in the ratio 40:60 v/v/ with microfiltered seawater. The data are the means of three independent experiments ± standard deviations (*n* = 3). ^*a*–*c*^Values of the same dataset with different superscript letters differ significantly (*p* < 0.05).

The highest CDM was found for the substrate obtained with Veron PS (2.92 ± 0.41 g/L), while the lowest value was observed in the substrate obtained after 6 h of pepsin treatment (2.17 ± 0.24 g/L) (Figure 3). Surprisingly, a high bioplastic amount was extracted from the substrate produced without enzymes (0.510 ± 0.028 g/L), and only a slightly higher amount characterized the substrate obtained with the pepsin treatment (0.526 ± 0.051 g/L). In details, the PY corresponded to 8.8 ± 0.8, 5.7 ± 0.5 and 8.5 ± 0.4 mg PHBV/g of wasted bread for P6, PS200 and NE, respectively. Although without significant differences, PC in biomass recovered from the substrate produced without enzymes (NE) was 19.76%, while it corresponded to 23.96% for the substrate obtained with the pepsin treatment (P6). The PC in biomass collected from the substrate treated with Veron PS (PS200) was significantly lower (11.81%).

According to these results, wasted bread substrate obtained without enzymes addition was employed for fermentation at 3 L-bioreactor level.

Under this condition, the CDM at the end of the process was 6.37 ± 0.92 g/L while the polymer collected (with the conventional extraction) corresponded to 1.529 ± 0.241 g/L. Purification with ethanol led to the recovery of 1.293 ± 0.216 g/L of the polymer (pPHBV). Similar values were found when PHBV was recovered from CDM using repeated washing with water (rPHBV, 1.487 ± 0.182 g/L) and then precipitated by adding ethanol (aPHBV, 1.187 ± 0.198 g/L).

The different procedures of extraction and purification led to PY of 21.6 ± 3.6, 24.8 ± 3.0, and 19.8 ± 3.3 mg PHAs/g of substrate for pPHBV, rPHBV, and aPHBV respectively.

### Poly(hydroxybutyrate-hydroxyvalerate) characterization

The sample extracted with the standard procedure (pPHBV) and the two samples extracted without the use of the CHCl_3_:H_2_O mixture (rPHBV and aPHBV, respectively not purified or purified through ethanol precipitation), were characterized.

Based on GC analysis, all samples were found to be characterized by a high PHBV content with a purity grade ranging from approximately 93% (w/w) to 100% (w/w), respectively for pPHBV/rPHBV and aPHBV (Table 2). GC analysis confirmed that samples corresponded to poly(hydroxybutyrate/hydroxyvalerate) (PHBV) copolymer with a HV content ranging from 10.78 ± 0.10 to 12.95 ± 0.04% w/w (Table 2). Above all, these results pinpoint that the adopted extraction procedure did not substantially affect the composition of extracted copolymer. However, slightly higher polymer purity and HV content were found in the aPHBV than in the other samples.

**TABLE 2:** Purity and composition of PHBV extracted from the wasted bread/seawater substrate with different methods (Figure 1): pPHBV, sample obtained from the CHCl_3_:H2O separation and the precipitation with cold ethanol; rPHBV, obtained by the replacement of the CHCl_3_:H_2_O separation with repeated washing with water; aPHBV, obtained by the replacement of the CHCl_3_:H_2_O separation with repeated washing with water, but followed by solubilization in CHCl_3_ and purification with cold ethanol.

Samples	PHBV purity (%, w/w)	HV content (%, w/w)
pPHBV	92.97 ± 1.78*^a^*	10.78 ± 0.10*^b^*
aPHBV	99.85 ± 0.15*^c^*	12.95 ± 0.04*^c^*
rPHBV	92.89 ± 0.67*^a^*	12.33 ± 0.14*^a^*

The data are the means of three independent experiments ± standard deviations (*n* = 3).

*^a–c^*Values in the same column with different superscript letters differ significantly (*p* < 0.05).

**FIGURE 4:**
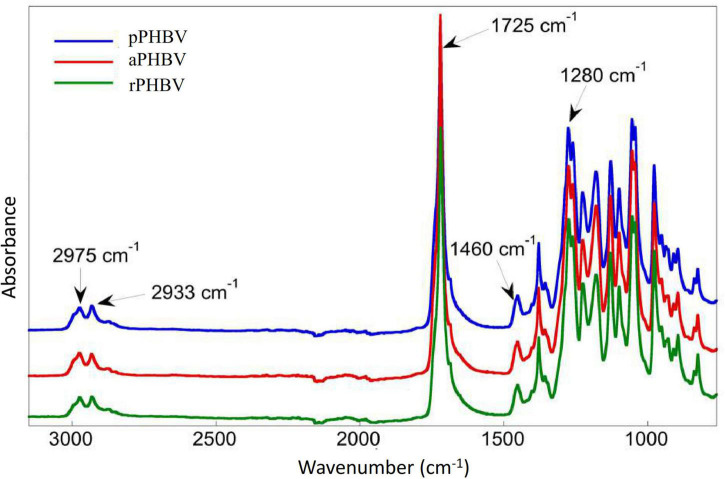
FT-IR spectra of PHBV produced in the wasted bread/seawater substrate: pPHBV, obtained from the CHCl_3_:H_2_O separation and the precipitation with cold ethanol; rPHBV, obtained by the replacement of the CHCl_3_:H_2_O separation with repeated washing with water; aPHBV, obtained by the replacement of the CHCl_3_:H_2_O separation with repeated washing with water, but followed by solubilization in CHCl_3_ and purification with cold ethanol.

The FT-IR spectra of the samples were superimposable, showing absorption of short-chain-length PHAs (scl-PHAs) between 2,975 and 2,933 cm^–1^ (CH, CH_2_, and CH_3_ stretching), at 1,725 cm^–1^ (ester C = O stretching), at 1,460 cm^–1^ (asymmetrical stretching of methyl group) and at 1,281 cm^–1^ and 1,227 cm^–1^ (C-O-C stretching) (Figure 4).

**FIGURE 5:**
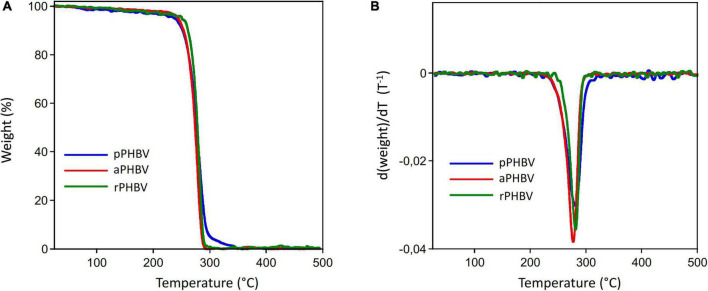
Thermogravimetric analysis curves of PHBV produced in the wasted bread/seawater substrate: pPHBV, obtained from the CHCl_3_:H_2_O separation and the precipitation with cold ethanol; rPHBV, obtained by the replacement of the CHCl_3_:H_2_O separation with repeated washing with water; aPHBV, obtained by the replacement of the CHCl_3_:H_2_O separation with repeated washing with water, followed by purification with cold ethanol. **(A)** Weight curve, **(B)** weight derivative as function of temperature.

Moreover, thermal stability and PHAs purity were investigated by TGA and differential TGA (DTGA), whose curves are reported in Figures 5A,B, respectively. The TGA curves of the analyzed samples showed a constant initial slight weight decrease up to about 210°C, presumably due to the decomposition of non-polymer biomass fraction or of impurities coming from the extraction process. Then, the complete decomposition of the PHAs samples occurred up to 320–340°C. In Table 3, the temperature of the polymer weight loss onset (T_*d*_*^onset^*) and of the maximum decomposition rate (T_*d*_*^max^*) as well of purity values obtained from TGA experiments are reported.

**TABLE 3:** Purity values (based on TGA) and thermal stability of PHBV extracted from the wasted bread/seawater substrate with different methods (Figure 1): pPHBV, sample obtained from the CHCl_3_:H2O separation and the precipitation with cold ethanol; rPHBV, obtained by the replacement of the CHCl_3_:H2O separation with repeated washing with water; aPHBV, obtained by the replacement of the CHCl_3_:H2O separation with repeated washing with water, but followed by solubilization in CHCl_3_ and purification with cold ethanol.

Sample	TGA purity (PHA %, w/w)	$T_{d}^{\mathrm{onset}}$ (°C)	$T_{d}^{\max}$ (°C)
pPHBV	96	232	282
aPHBV	98	225	276
rPHBV	96	248	280

**FIGURE 6:**
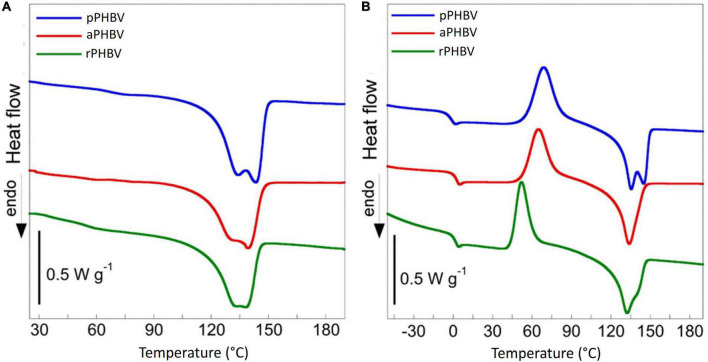
Differential scanning calorimetry thermograms of PHBV produced in the wasted bread/seawater substrate: pPHBV, obtained from the CHCl_3_:H_2_O separation and the precipitation with cold ethanol; rPHBV, obtained by the replacement of the CHCl_3_:H_2_O separation with repeated washing with water; aPHBV, obtained by the replacement of the CHCl_3_:H_2_O separation with repeated washing with water but followed by solubilization in CHCl_3_ and purification with cold ethanol. **(A)** First heating scan from 25 to 190°C at 10°C min^–1^, **(B)** second heating scan from −70 to 190°C at 10°C min^–1^. The DSC curves were vertically shifted for sake of clarity.

The differential scanning calorimetry (DSC) allowed to evaluate the thermal behavior of PHBV samples. The thermograms obtained in the first and the second heating scans are displayed in Figure 6 and the thermal properties of the samples are reported in Table 4. Melting of the polymers in the 100–150°C temperature range is the only transition occurring in the first heating scan (Figure 6A). The presence of a double endothermic peak is quite usual in PHB and PHBV copolymer and is due to the melting of original crystalline lamellae which, during the heating, recrystallize in more ordered morphology that melts at higher temperature or to the presence of original crystallites with different thickness. During the cooling, carried out at 30°C/min (data not shown), the polymers did not crystallize and were completely amorphous in the subsequent second heating scan. In fact, the thermograms reported in Figure 6B shows the presence of specific heat capacity change (ΔC_*p*_) at the glass transition temperature (T_*g*_) as well as cold crystallization (T_*cc*_) and melting process (T_*m*_) characterized by the same specific enthalpy (ΔH_*cc*_ and ΔH_*m*_, respectively) (Table 4). The thermal behavior of the three samples showed small but significant differences, attributable neither to polymer composition nor to purity, since they were almost equal. Besides a lightly lower glass transition temperature, pPHBV displayed a higher ΔH_*m*_ value, related to the sample crystallinity, respect with the other two samples, as in the first run.

**TABLE 4:** Thermal properties of PHBV extracted from the wasted bread/seawater substrate with different methods (Figure 1): pPHBV, obtained from the CHCl_3_:H_2_O separation and the precipitation with cold ethanol; rPHBV, obtained by the replacement of the CHCl_3_:H_2_O separation with repeated washing with water; aPHBV, obtained by the replacement of the CHCl_3_:H_2_O separation with repeated washing with water, but followed by solubilization in CHCl_3_ and purification with cold ethanol.

Sample	1st heat		2nd heat					
	T_m_ (°C)	Δ H_m_ (J g^–1^)	T_g_ (°C)	Δ Cp (J K^–1^ g^–1^)	T_cc_ (°C)	Δ H_cc_ (J g^–1^)	T_m_ (°C)	Δ H_m_ (J g^–1^)
pPHBV	133; 143	68	−3	0.53	69	57	135; 145	56
aPHBV	129; 139	58	1	0.54	65	49	134	49
rPHBV	132; 138	56	1	0.52	52	50	132	50

## Discussion

It has been estimated that the bread wasted daily worldwide is around hundreds of tons (Melikoglu and Webb, 2013). The loss of bread occurs through the entire supply chain, from the bakery (for example the crust removed from sandwich bread, that can reach 40% of the entire production) (Verni et al., 2020), to the distribution network and at household level. Nowadays, most of the leftover bread is disposed as waste and only a little amount of bread is reused, mainly as feed (Verni et al., 2022). Different attempts for wasted bread valorization include the production of food ingredients, like glucose syrup (Kwan et al., 2018; Riaukaite et al., 2019), or substrate for brewing (Dlusskaya et al., 2008; Brancoli et al., 2020) or the production of functional ingredients for the bakery sector (Immonen et al., 2020; Nionelli et al., 2020; Verni et al., 2022).

Aiming at developing zero-waste strategies for the long time sustainability of the food chains, it is also necessary to consider that a large part of the wasted bread cannot be reused for food or feed use, due to the rapid microbial deterioration and the inherent sensory and safety issues. Therefore, there is the necessity to identify novel solutions aimed at the upcycle of the not more edible wasted bread with the production of added-value innovative products to be reintroduced into the market. For example, the production of bread-based multi-functional soil amendments with high density of viable plant growth-promoting microorganisms, has been recently proposed (Cacace et al., 2022).

Based on these premises, this research aims at investigating the use of wasted bread for the production of completely biodegradable bioplastic (Han et al., 2017) through the use of the PHAs-synthesizing microorganism *Hfx. mediterranei* and seawater.

PHAs have been identified as promising candidates for bio-based plastics production thanks to suitable and combined biodegradability, biocompatibility, and thermoplasticity properties (Han et al., 2017; Amaro et al., 2019). Nevertheless, the PHAs large-scale production is still limited by high production costs (Albuquerque and Malafaia, 2018), that can be reduced at different levels, including the choice of a cheap and proper substrate and suitable pre-treatments and extraction/purification processes. Indeed, as an example, the cost of the feedstock is estimated to account for about 40–50% of the total production costs (Wang et al., 2021), Therefore, the use of wastes from the agri-food chains is considered another important prerequisite for the set-up of sustainable processes for bioplastic production (Raho et al., 2020). Moreover, the use of halophiles could offer an efficient solution for bioplastic production, mainly thanks to their large adaptability to the extreme conditions. Indeed, the high salinity requirements reduces the potential microbial contamination (Kourmentza et al., 2017), avoiding expensive sterilization pre-treatments of the waste substrates.

Wasted bread includes a high quantity of starch (over 70% on dry matter) and proteins (up to 14% on dry matter) (Melikoglu and Webb, 2013). A preliminary assay was performed to identify a suitable ratio bread:water for the substrate production. As expected, a relevant amount of carbohydrates was found after homogenization and incubation of bread in water alone. Although baking led to the inactivation of endogenous flour enzymes, it is well known that pre-gelatinized starch, which characterize bread, presents higher swelling power and solubility in cold water than native starch (Ma et al., 2022), probably due to the weakening of intramolecular hydrogen bonds and the decrease in the interaction between amylose and amylopectin molecules and between amylopectin chains (Ma et al., 2022).

Nevertheless, according to several reports that already demonstrated the effectiveness of bioprocessing treatments of wasted bread for the production of medium suitable for the microbial growth (Simpson et al., 2012; Verni et al., 2020), the effect of enzymatic treatments was also investigated. A starting formulation of the bread-derived substrate was indeed developed with the use of proteolytic and amylolytic enzymes, whose use aimed at increasing the carbohydrates concentration in the medium. In particular, the expected outcomes for the use of enzymes (singly or pooled) were i) the promotion of the release of the starch trapped in the protein network (proteases) and ii) the hydrolysis of the starch into derivatives (e.g., dextrins, malto-dextrins, maltose, glucose) more easily used by the microorganism.

In response to the exponential growth of enzymes use in biotech industries, impressive advancements and impact of enzyme technology occurred during recent years. Microbial enzymes, in particular, are largely employed at industrial level due to high yields, activity, reproducibility, cost-effective production, use of inexpensive media, easy optimization, and strain improvement, that, together with efficient downstream processing and immobilization strategies, have immensely helped in decreasing the cost of the commercial preparations (Fasim et al., 2021). In this work, the first set-up of the substrates included the evaluation of the effect of different enzymatic treatments carried out with both laboratory-grade preparations and a commercial formulation intended for industrial uses.

It must be underlined that, potentially, starch could be directly metabolized by *Hfx. mediterranei* without the need of an intense enzymatic hydrolysis of the bread-derived substrate (Rodriguez-Valera and Lillo, 1992; Huang et al., 2006; Yanti et al., 2021). Morevover, *Hfx. mediterranei* was previously characterized for the production and release of α-amylase in a starch-enriched medium (Pérez-Pomares et al., 2003). Nevertheless, bread starch is subjected to an intense thermal treatment (baking), that modifies its native structure, and its availability can be also affected by the gluten network presence. Moreover, based on our knowledge, there are few information regarding the preferential use of carbohydrates and the effect on growth of bioplastic synthesis, thus making necessary the investigation of the enzymatic treatment effects.

Another factor investigated in the set-up of the bread-derived substrate was the supplementation in mineral salts. The previous literature has already demonstrated the need to supplement the growth substrate of *Haloferax* spp. with highly concentrated salts (Koller et al., 2007; Koller, 2019). The salt concentration suitable for the growth phase ranges from 100 to 300 g/L, while concentrations between 160 and 250 g/L are associated to high production of PHBV (Koller, 2019). In particular, the presence of minerals other than sodium and chlorine is needed. Magnesium, in particular, represents one of the most important minerals for *Hfx. mediterranei* growth (Torreblanca et al., 1986), while the phosphorous limitation was considered essential for increasing the PHBV accumulation (Lillo and Rodriguez-Valera, 1990) with the optimal concentration evaluated by Melanie et al. (2018). In this work, we demonstrate the possibility to use seawater as a direct supplement to the substrate, focusing on its wide availability and its low cost. Seawater generally contains high amount (from 0.8 to 2 g/L) of magnesium, calcium, and potassium (Bardi, 2010; Barbarisi et al., 2019; De Bellis et al., 2020). Although East Mediterranean sea, which includes Italian coasts, is described for the limited phosphorus availability (Feingersch et al., 2010), its concentration in the substrate used for *Hfx. mediterranei* growth was not determined in this study.

Overall, the use of the protease pepsin, and of a sequential treatment with α-amylase, gave the better results in terms of carbohydrates release. The use of the Veron PS enzyme gave results only slightly less efficient. Regarding the seawater supplementation, it was found that when used at very high percentage (60% of the total substrate volume) the growth of the microorganism was optimal.

CDM and the amount of recovered PHBV observed in bread/seawater substrate are similar to those previously reported in literature and obtained, for example, using seaweed hydrolysate (Ghosh et al., 2019) and optimized media (Sato et al., 2021). However, higher CDM and PHBV productivity values were also reported in literature, for example for substrates obtained with pre-treated whey lactose (Koller et al., 2005, 2007) and vinasse (Bhattacharyya et al., 2012). Although the growth (CDM) of the microorganism was significantly higher on the substrates produced with the enzymatic treatments, statistically comparable polymer content and yield (PC and PY) were obtained by using the substrate made with untreated bread and seawater, thus helping the reduction of the production costs.

It has been previously reported that the highest bioplastic synthesis did not always correspond to the highest rate of cell biomass production (Cui et al., 2017; Ferre-Guell and Winterburn, 2017). Indeed, different factors affect the accumulation of biomass or intracellular content of PHBV, thus making necessary the evaluation of both parameters. The effect of different macronutrients (e.g., glucose, amino acids) and micronutrients (e.g., minerals) showed different results in terms of CDM and PHBV production (Sato et al., 2021). Salinity is moreover recognized by cause of different response of the microorganism in growth and PHAs synthesis (Pacholak et al., 2021). Cui et al. (2017) already found that the condition (salinity, in particular) that promoted the highest synthesis of PHAs corresponded to that resulting in 70% of potentially achievable CDM.

Therefore, considering the copolymer content (PC), i.e., the PHBV accumulated into the biomass, the ratio of copolymer synthesized from the substrate (PY) and the research of an efficient protocol with low external and expensive inputs (mainly related to the enzymes cost), the wasted bread media prepared without the use of enzymes was tested in 3 L-bioreactor. Under bioreactor conditions, characterized by constant pH, temperature, and stirring, the PHBV production resulted higher than that previously found in lactose-hydrolysed whey (Raho et al., 2020), while higher PHBV and similar CDM was found in 3 L bioreactor using also continuous feeding with C4:0/C5:0 fatty acids, constant oxygenation in fermentations lasted more than 11 days (Gonzalez and Winterburn, 2022).

Several studies reported that *Hfx. mediterranei* is capable to produce PHBV copolymers up to 60–65% of the biomass efficiently utilizing agro-industrial residues (Koller et al., 2017; Stanley et al., 2021). Nevertheless, polymer content lower than 20% of the CDM, like those found in this work, were often reported (Melanie et al., 2018; Stanley et al., 2021) since culture conditions and nutrient supplies seriously affect the PHAs accumulation capacity of the microorganism.

One of the advantages of the halophiles use, is that microbial cells can be easily lysed in normal water due to the high intracellular osmotic pressure; thus, further reducing PHAs recovery cost related to solvents (Quillaguamán et al., 2010), like chloroform, also associated to environmental issues.

In this work, together with the conventional extraction procedure, including the CHCl_3_:H_2_O use, a water-based extraction protocol for PHBV was tested. In particular, the water-based extraction procedure was followed (aPHBV) or not (rPHBV) by chloroform solubilization and ethanol precipitation. Polymer yield was the highest for rPHBV.

The GC analysis showed similar values in terms of purity and HV contents among all the three extracted samples. This outcome is particularly interesting since it highlights the possibility to obtain a very high-purity polymer also without the chloroform and ethanol purification procedure (rPHBV sample vs pPHBV and aPHBV). In all samples the HV content in the stored polymer was, as expected, similar as mainly depending on the composition of the used feedstock and the microorganism used. The HV is synthesized mainly in presence of precursors, like valeric or propionic acid (Policastro et al., 2021). This latter is commonly present in wasted bread, since commonly added as antimould preservative (Coda et al., 2008; Khaldun et al., 2010) or as result of lactic acid bacteria synthesis (Özcelik et al., 2016). Lactic acid bacteria are largely present in Type 0, -I or -II-sourdough, commonly employed for breadmaking at industrial and artisanal level (De Vuyst et al., 2021). Nevertheless, several authors reported the build-up of PHBV from structurally unrelated carbon sources (e.g., extruded starch) in *Hfx. mediterranei* without requiring particular precursors (Kulkarni et al., 2010). This provides a competitive advantage over other PHAs-producing bacteria, since propionic acid and valeric acid are costly and sometimes affecting the cells viability (Ferre-Guell and Winterburn, 2017).

However, the conventional extraction/purification procedure adopted for obtaining pPHBV led to the recovery of a copolymer with a slightly lower ratio of HV. The HV content impacts the thermal and mechanical properties of the stored polymer and, in turn, its final application, as widely reported in the literature (Melendez-Rodriguez et al., 2021).

The spectra of PHBV by FT-IR spectroscopy showed all the expected absorption of scl-PHAs (Salgaonkar and Bragança, 2017; Roja et al., 2019), while the absence of absorption bands between 1,680 and 1,480 cm^–1^ showed that no detectable protein residues from biomass are observed. PHAs degradation usually takes place by a random chain scission mechanism in the 250–300°C temperature range (Hahn and Chang, 1995) with a single weight loss step from which it is possible to determine the sample purity.

The purity values obtained from TGA experiments are fully in agreement with the GC results, whereby all samples present a high purity. Also, all analyzed samples show a slightly lower thermal stability with respect to the stability typically presented by PHBV copolymers with similar monomeric compositions. Due to the high purity grade of the samples, the observed lower thermal stability cannot be due to the presence of impurities that act as catalysts for the thermodegradation of PHAs, as reported in the literature (Hilliou et al., 2016), and it requires further investigation. By comparing the melting enthalpy reported for DSC analysis, a greater crystallinity of pPHBV was observed with respect to that of the other two samples. This finding could be correlated to the lower HV content, with a positive effect on the biodegradability of the bioplastic (Mergaert et al., 1992; David et al., 2019). However, it could be also due to the different treatments to which the samples were subjected in the extraction process. Moreover, the possible presence of impurities with plasticizing effect in rPHBV and aPHBV could justify these differences. Furthermore, it can be supposed that the significant lower cold-crystallization temperature (T_*cc*_) of the impurified sample is due to biomass solid debris that acted as a nucleating agent.

## Conclusions and prospects

To the best of our knowledge, this is the first research demonstrating the suitability of wasted bread, supplemented with seawater, to be used as substrate for bioplastic production through fermentation. Results demonstrated that a solvent-free extraction, exclusively based on osmotic shock, could be used to recover the bioplastic from cells. On the basis of the results collected, it can be assumed that 1 kg of PHBV, moreover characterized by high purity grade and a HV content that confers good thermoplastic properties, can be produced from less than 50 kg of wasted bread. The proposed protocol already represents an effective alternative for not-more-edible waste bread valorization, considering that the huge disposal of wasted bread as common waste correspond to high economic loss and often leads to environmental issue. Nevertheless, further research focused on the optimization of the proposed fermentation protocol could lead to improvement of in the bioplastic yield, rendering more sustainable and convenient the conversion.

## Data availability statement

The raw data supporting the conclusions of this article will be made available by the authors, without undue reservation.

## Author contributions

MM was responsible for formal analysis, data curation, investigation, and writing the original draft. GS and SA were responsible for formal analysis. AM and MiV were responsible for data curation, investigation, and for manuscript revision. EP was responsible for resources, funding acquisition, manuscript revision. MaV was responsible for the conceptualization of the study, investigation, validation, and writing. CR was responsible for the conceptualization of the study, investigation, validation, writing, reviewing, and editing. All authors contributed to the article and approved the submitted version.
